# The Neural Processing of Vocal Emotion After Hearing Reconstruction in Prelingual Deaf Children: A Functional Near-Infrared Spectroscopy Brain Imaging Study

**DOI:** 10.3389/fnins.2021.705741

**Published:** 2021-07-28

**Authors:** Yuyang Wang, Lili Liu, Ying Zhang, Chaogang Wei, Tianyu Xin, Qiang He, Xinlin Hou, Yuhe Liu

**Affiliations:** ^1^Department of Otolaryngology, Head and Neck Surgery, Peking University First Hospital, Beijing, China; ^2^Department of Pediatrics, Peking University First Hospital, Beijing, China; ^3^Department of Otolaryngology, Head and Neck Surgery, The Second Hospital of Hebei Medical University, Shijiazhuang, China

**Keywords:** vocal emotion, cochlear implant, prelingual deaf, infant and toddler, functional near-infrared spectroscopy

## Abstract

As elucidated by prior research, children with hearing loss have impaired vocal emotion recognition compared with their normal-hearing peers. Cochlear implants (CIs) have achieved significant success in facilitating hearing and speech abilities for people with severe-to-profound sensorineural hearing loss. However, due to the current limitations in neuroimaging tools, existing research has been unable to detail the neural processing for perception and the recognition of vocal emotions during early stage CI use in infant and toddler CI users (ITCI). In the present study, functional near-infrared spectroscopy (fNIRS) imaging was employed during preoperative and postoperative tests to describe the early neural processing of perception in prelingual deaf ITCIs and their recognition of four vocal emotions (fear, anger, happiness, and neutral). The results revealed that the cortical response elicited by vocal emotional stimulation on the left pre-motor and supplementary motor area (pre-SMA), right middle temporal gyrus (MTG), and right superior temporal gyrus (STG) were significantly different between preoperative and postoperative tests. These findings indicate differences between the preoperative and postoperative neural processing associated with vocal emotional stimulation. Further results revealed that the recognition of vocal emotional stimuli appeared in the right supramarginal gyrus (SMG) after CI implantation, and the response elicited by fear was significantly greater than the response elicited by anger, indicating a negative bias. These findings indicate that the development of emotional bias and the development of emotional perception and recognition capabilities in ITCIs occur on a different timeline and involve different neural processing from those in normal-hearing peers. To assess the speech perception and production abilities, the Infant-Toddler Meaningful Auditory Integration Scale (IT-MAIS) and Speech Intelligibility Rating (SIR) were used. The results revealed no significant differences between preoperative and postoperative tests. Finally, the correlates of the neurobehavioral results were investigated, and the results demonstrated that the preoperative response of the right SMG to anger stimuli was significantly and positively correlated with the evaluation of postoperative behavioral outcomes. And the postoperative response of the right SMG to anger stimuli was significantly and negatively correlated with the evaluation of postoperative behavioral outcomes.

## Introduction

Emotional communication is a universal form of expression that enables people to overcome language and cultural barriers (Thompson and Balkwill, 2006; Bryant and Barrett, 2008; Pell et al., 2009). Deficits in vocal emotion perception (facial expressions are not always visible in many environments) not only affect the quality of life and social interactions among adults but also potentially affect the cognitive and social development of infants and toddlers (Denham et al., 1990). Responding to emotional stimuli is one of the first developmental milestones, and has higher credibility in infants and toddlers, who have immaturely perception and cognition. Meanwhile, in other research, newborns have been shown to have a preference for the infant-directed speech of female voices, which can be attributed to infant-directed speech generally conveying more emotion and information, compared with the suppressed emotion expression in most adult communication (Glenn and Cunningham, 1983; Cooper and Aslin, 1989; Pegg et al., 1992). These findings revealed that the perception and recognition of vocal emotions are significant factors during cognition and communication in early life. Numerous studies have shown that establishing a strong emotional connection with adults is crucial for the physical and intellectual development of infants and toddlers (Ainsworth and Bell, 1970; Drotar and Sturm, 1988). The perception of vocal emotion is likely to be a more significant factor during early infant development because the auditory system develops earlier than the visual system (Gottlieb, 1971; Kasatkin, 1972). However, infants and toddlers with hearing loss face difficulties in emotional communications (Ludlow et al., 2010; Most and Michaelis, 2012).

Many studies have examined the neural processing for vocal emotions in infants with normal hearing, which may provide a reference for the neural processing associated with hearing loss. Information regarding the neural processing mechanism of vocal emotion was also reported in neonates (Zhang et al., 2017, 2019; Zhao et al., 2019). The results showed that vocal emotions enhance the activation of the right superior temporal gyrus (STG), the left superior frontal gyrus (SFG), and the left angular gyrus, which may indicate the regions associated with the neural processing of vocal emotional perception during neonatal development. Moreover, happiness elicited increased neural responses in the right STG and the right inferior frontal gyrus (IFG) as compared to fear and anger, and these results also indicate that neonates’ response to positive vocal emotions takes precedence over negative vocal emotions, which is contrary to the negative biases observed in adult studies. Up to now, very little is known about the neural processes of vocal emotion in infants and toddlers with normal hearing. Studies have shown that infants younger than 6 months show a preferential response to positive emotions received through vocal emotional stimuli (Farroni et al., 2007; Vaish et al., 2008; Rigato et al., 2010). This result is supported by Trainor et al. (2000), who demonstrated that infants are more inclined to listen to infant-directed speech associated with positive emotions than to infant-direct speech associated with negative emotions. Conversely, negative bias refers to the priority processing of negative information in the brain, which typically appears in infants between 6 and 12 months of age (Peltola et al., 2009; Grossmann et al., 2010; Hoehl and Striano, 2010). The findings of these previous studies seem to indicate that emotional preferences are different at different stages of human development.

Emotions are expressed through a combination of numerous acoustic cues (Linnankoski et al., 2005; Sauter et al., 2010). Hearing loss will affect the perception and recognition of emotions in speech, including cochlear implant (CI) users (Most et al., 1993; Ludlow et al., 2010; Wiefferink et al., 2013; Chatterjee et al., 2015), because the reduction of acquired acoustic cues makes it difficult to distinguish acoustic emotions. Speech processing and adaptive behaviors in the social environment depend on the effective decoding of vocal emotions (Fruhholz and Grandjean, 2013), especially for deaf infants and toddlers who cannot recognize words and sentences (Eisenberg et al., 2010). Therefore, hearing loss in infants and toddlers can have long-term impacts on language learning, social functioning and psychological development (Wauters and Knoors, 2008; Schorr et al., 2009; Eisenberg et al., 2010; Geers et al., 2013). Previous studies have concluded that the auditory emotion recognition of children with severe-to-profound hearing loss is significantly worse than that of chronological- and mental-age-matched normal hearing children (Ludlow et al., 2010). However, no significant difference in auditory emotion recognition has been observed between children with mild-to-moderate hearing loss relative to their normal-hearing peers (Cannon and Chatterjee, 2019). Infants and toddlers with congenital severe-to-profound hearing loss represent good models for studying emotional recognition processes after hearing reconstruction. Observing differences in the perception and recognition of vocal emotion between children with congenital severe-to-profound hearing loss and normal-hearing children will help us to better understand the development of vocal emotion recognition during the early stages of life. However, little is known about the effects of severe-to-profound hearing loss and hearing reconstruction on the neural processing of vocal emotion in infants and toddlers.

To investigate these phenomena, neural data from early life stages should be included. To date, no brain imaging studies have focused on the vocal emotion processing that occurs in infants and toddlers with severe-to-profound hearing loss who received few vocal emotional cues from the social environment. The auditory perception of infants and toddlers with hearing loss can be reconstructed through the use of cochlear implants (CIs) (Zeng et al., 2008). The use of CIs is a world-recognized method for hearing reconstruction in individuals with severe-to-profound sensorineural hearing loss. The use of CI has significantly benefited children with severe to profound hearing loss in acoustic emotion recognition (Chatterjee et al., 2015). However there are still some imperfections in the sound collection, information processing and transmission of existing cochlear implant systems, such as the transmission of spectro-temporal fine structure information are degraded (Xu et al., 2009; Kang et al., 2010; Kong et al., 2011). The acoustic information received by CI users is decreased. Therefore, perceiving prosodic information is difficult for CI users compared to people with normal hearing (such as vocal emotion, music appreciation) (Jiam and Limb, 2020). And prosodic limitation will bring adverse consequences to the decoding and communication of vocal emotions for CI users (Planalp, 1996). But the neural mechanism of how CI users processing this severely reduced acoustic information after auditory cortex deprivation and remodeling remains unclear. For example, if the incomplete acoustic information can enable a specific emotional state to reproduce the experience in the sensory-motor system, this may involve embodied cognition theory (Damasio, 1989; Wilson, 2002; Niedenthal, 2007). In addition, the correlation between cortical activation elicited by these vocal emotional stimuli and postoperative behavioral outcomes remains to be studied. Part of the problem is the lack of neuroimaging tools suitable for early, repeatable measurements. Prelingual deaf infant and toddler CI users (ITCIs) represent a unique group of people who have experienced hearing loss and reconstruction, with limited hearing experience. Research on this group will bring new perspectives and supplement existing knowledge in this field.

In this study, we used functional near-infrared spectroscopy (fNIRS) to observe changes between the preoperative and early postoperative neural response to four vocal emotions in prelingual deaf ITCIs. The primary advantages of fNIRS include low invasiveness and the characteristics of optical principles, which are compatible with ITCI and allow for the safe and repeated use of fNIRS. In addition, the ability to use fNIRS without generating additional noise makes this a compatible evaluation modality for auditory tasks. fNIRS also offers higher spatial resolution than event-related potentials and can be easily adapted to accommodate the test subject’s head and body movements (Piper et al., 2014; Saliba et al., 2016). The ability to collect data from low-attention and active infants and toddlers was further enhanced by the use of fNIRS. We tested the participants within 1 week prior to the CI operation (preoperative test) and within 1 week after turning the CI on (postoperative test). We presented four vocal emotional stimuli for the subjects, including fear (negative), anger (negative), happiness (positive), and neutral. The Infant-Toddler Meaningful Auditory Integration Scale (IT-MAIS)/MAIS (Zimmerman-Phillips et al., 1997) and Speech Intelligibility Rating (SIR) scales (Allen et al., 1998) were used to evaluate the participants’ speech perception and expression abilities. This design allowed for three critical questions to be addressed. (1). How the neural processing (regions and intensity of cortical activation) for vocal emotional stimuli in prelingual deaf ITCI differ between preoperative and postoperative tests. (2). Whether vocal emotion recognition and bias exist in the processing of prelingual deaf ITCIs during preoperative and postoperative tests and whether these change following CI use (see Zhang et al., 2017, 2019). (3). Whether any correlations exist between the neural responses to vocal emotional stimuli and speech perception in prelingual deaf children and their expression abilities and how these differ between the preoperative and postoperative tests.

Although we make hypotheses based on the results of studies performed in people with normal hearing, our research is exploratory due to the lack of existing brain imaging studies evaluating the effects of severe-to-profound hearing loss on vocal emotional processing patterns. We hypothesized that the neural responses to the four vocal emotions in the preoperative test results of prelingual deaf ITCIs would not differ significantly due to the impacts of hearing loss, whereas the neural processing will differ from that observed for normal-hearing infants (Zhang et al., 2019). That is, the region and intensity of cortical activation should be different. The correlation between neural and behavioral results will be difficult to observe at this time due to the lack of sufficiently mature auditory cortex development. The hypothesis for postoperative test results was that differences would be observed in vocal emotion recognition ability and neural processing relative to those in the preoperative test. After the hearing is reconstructed, the auditory cortex receives better auditory stimulation. Because emotion recognition and positive bias could be observed in neonates as early as 0–4 days after birth in previous studies (Zhang et al., 2019), we expected that ITCIs would respond to the four vocal emotional stimuli in 0–7 days after the CI is activated, and a stronger neural response will be observed in the right temporal area, with a positive bias. That is, happiness will cause stronger activation of the cortex than other vocal emotions. However, fewer emotions will be recognized due to the limitation of CI itself, and the cortical activation region elicited by vocal emotional stimulation will be different from that of neonates with normal hearing, and the activating intensity will be weaker. We don’t expect to find significant differences in preoperative and postoperative behavioral tests, but that overall performance on these assessments was being examined to determine if these common clinical assessments would be correlated with cortical activation. And we expected to observe a correlation between the neural response elicited by vocal emotional stimuli in the right temporal region and behavioral results at this time because the correlation between cortical activation elicited by auditory and visual stimulation and behavioral results has been observed in previous deaf adult studies (Anderson et al., 2019). We hope that these results can establish a baseline for future research in this field.

## Materials and Methods

### Participants

Twenty-two prelingual deaf infants and toddlers with bilateral severe-to-profound sensorineural hearing loss (10 girls; age: 13–38 months, mean: 22.5 ± 6.43 months) participated in this study (see Table 1 for detailed information). All participants failed the first hearing screening of the otoacoustic emissions test combined with automatic auditory brainstem response 3–5 days after birth and failed the second hearing screening performed within 42 days of birth. All participants met the Chinese national guidelines for cochlear implantation (Editorial Board of Chinese Journal of Otorhinolaryngology Head and Neck Surgery et al., 2014). Before implantation, all participants were evaluated for auditory ability: The click auditory brainstem response thresholds for both ears were above 90 dB nHL; and the auditory steady-state response was above 90 dB nHL in response to frequency thresholds of 2 kHz and above. Cooperating with a pure tone audiometry test or auditory word recognition test is difficult for the participants in this study because our participants lack sufficient auditory and speech skills and attention to understand and perform the test procedure. Therefore, they were assessed by unaided pediatric behavioral auditory testing (PBA), using thresholds above 80 dB HL, and aided pediatric behavioral auditory testing, using thresholds above 60 dB HL, for both ears. Participants also met the following criteria: (A) except for hearing, other physiological development was normal (including vision); (B) imaging examinations showed no deformities of the inner ear; (C) the postoperative X-ray examination did not reveal any abnormalities, and implanted electrodes were correctly located in the cochlea; (D) all impedances for all electrodes after surgery are within the normal range; (E) the clinical team evaluated the comfort and threshold levels of each electrode position according to standard clinical protocols; and (F) the participants were clinically asymptomatic (No abnormality or discomfort) at the two times of fNIRS recording.

**TABLE 1:** Demographic and clinical information of participants.

Subject	Gender	Implanted-age (months)	Implantation side	Cochlear implant	Preoperative aided thresholds (Implanted side)	Preoperative aided thresholds (contralateral side)
1	Girl	23	Right	Nucleus CI-512	80	90
2	Girl	17	Right	Nucleus CI-512	70	60
3	Boy	21	Right	Nucleus CI-512	66	94
4	Girl	30	Right	Nucleus CI-512	100	100
5	Boy	34	Right	Nucleus CI-512	70	70
6	Boy	17	Right	Nucleus CI-512	65	65
7	Boy	16	Right	Nucleus CI-512	90	100
8	Boy	27	Right	Nucleus CI-512	70	80
9	Girl	16	Right	Nucleus CI-512	95	95
10	Boy	22	Right	Nucleus CI-512	98	98
11	Girl	23	Right	Nucleus CI-512	96	99
12	Girl	28	Right	Nucleus CI-512	94	96
13	Boy	15	Right	Nucleus CI-512	79	65
14	Boy	13	Right	Nucleus CI-512	71	86
15	Girl	16	Right	Nucleus CI-512	95	95
16	Girl	16	Right	Nucleus CI-512	100	100
17	Boy	38	Left	Nucleus CI-512	70	65
18	Girl	27	Left	Nucleus CI-512	70	75
19	Boy	25	Right	Nucleus CI-512	80	80
20	Boy	25	Right	Nucleus CI-512	90	90
21	Boy	26	Right	Nucleus CI-512	100	100
22	Girl	20	Right	Nucleus CI-512	60	60
Mean (SD)		22.5(6.43)			82.23(13.66)	84.68(14.70)

### Behavioral Measurements

At every visit, participants’ auditory perception was assessed by the IT-MAIS/MAIS (Zimmerman-Phillips et al., 1997), and speech production was assessed by the SIR (Allen et al., 1998). These two tests reported by caregiver were widely used tools for screening and monitoring the hearing and speech development of infants and toddlers (Cavicchiolo et al., 2018; Lu and Qin, 2018; Yang et al., 2020). The IT-MAIS/MAIS assessment of auditory perception in infants and young children includes three dimensions: Adaptability to CI, auditory perception ability, and auditory recognition ability. The use of the IT-MAIS/MAIS enabled us to perform a more comprehensive assessment and monitoring of auditory development in infants and toddlers at earlier stages. To better measure the participant’s hearing and speech abilities, we converted the scores of all assessments to a 0–100 scale. The total score of ITMAIS/MAIS is 40 points. We multiply the score by 2.5. There are 5 levels of SIR, and each level counts as 20 points.

### fNIRS Experimental Stimuli and Procedure

**FIGURE 1:**
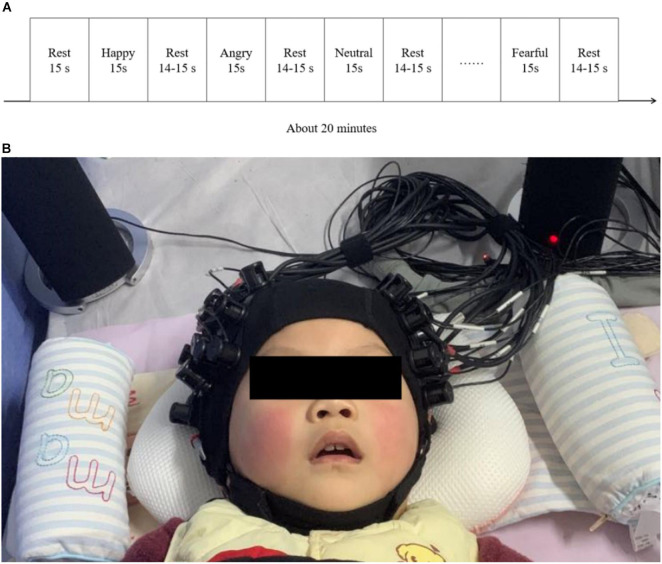
fNIRS test procedures and environment. **(A)** A schematic representation of the block design used for the vocal emotional stimulation experimental procedure. **(B)** An example of the positioning of the participants during the fNIRS test. The test was conducted in a dark environment, and the lights in the room were switched off.

The auditory stimuli program presented to ITCI users was generated by E-Prime (version 2.0, Psychology Software Tools, United States). Vocal emotional stimulation included four types: Fear, anger, happiness, and neutral. The vocal stimulation materials used in this study were obtained from the Chinese vocal emotional stimulation material database (Liu and Pell, 2012). We used infant-directed vocal emotional stimulation as the stimulus material because this stimulus does not require substantial auditory experience, and vocal emotional perception develops earlier than speech perception capabilities and has been shown to be effective even in neonates (Zhang et al., 2017, 2019). These vocal emotional stimuli are composed of pseudo-sentences, which are non-semantic but contain grammatical information and deliver vocal emotional information at the same time. Stimulation materials were read in infant-directed speech prosody by a native Chinese female. The recognition rate and emotional intensity of vocal emotional stimuli have been confirmed in our previous research (Zhang et al., 2019). Vocal emotional stimulation is presented in a block design, with 15-s sound presentation blocks interleaved with silent blocks of varying durations between 14 and 15 s. The four types of emotional prosodies were presented in random order. Each type of emotional prosody was repeated 10 times, and the entire stimulation program lasts approximately 20 min (Figure 1A).

Preoperative brain imaging using fNIRS was performed after the participant agreed to receive CI but before the operation when the participant was asleep. During the preoperative test, participants wore hearing aids on both sides during the test. Participants visited the hospital approximately 4 weeks after surgery to activate the CI, and fNIRS brain imaging was performed within 0–7 days after CI activation (mean: 5.727 ± 2.700 days). fNIRS were conducted in the hearing room of the Department of Otolaryngology Head and Neck Surgery, Peking University First Hospital. During the postoperative test, participants wore both CI and hearing aids. The sound was passively presented through a pair of speakers (Edifier R26T, Edifier, China). The speakers were placed 10 cm from the left and right ears of the participants (Figure 1B). To reflect the typical level of conversational speech, the sound pressure level was maintained at 60–65 dB SPL. To ensure the collection of fNIRS data, extra optical and acoustic signals were excluded to the greatest extent possible during the experiment, and the average background noise intensity was measured near 30 dB SPL. These data were measured in the listening position using a sound level meter (HS5633T, Hengsheng, China) during the experiment and in a quiet state, with participants absent. The fNIRS recording was performed with the participants in a quiet sleeping state, or in a quiet state of alert (see Cheng et al., 2012; Gomez et al., 2014; Zhang et al., 2019; Figure 1B). We recruited 30 ITCIs. ITCIs who started crying or were too active during fNIRS recording were not included in the analysis. Therefore, the final analysis was performed on a data set that included 22 participants.

### fNIRS Data Recording

**FIGURE 2:**
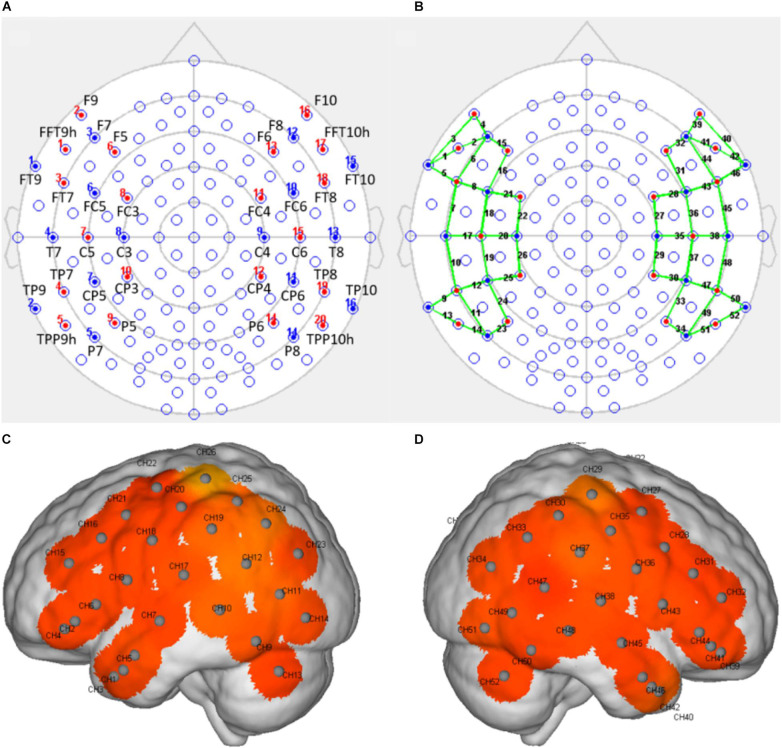
The arrangement of the light sources and detectors and the positions of the detection channels. **(A)** The position of the light sources and detectors, arranged according to the 10–20 system, with red indicating a light source and blue indicating a detector. **(B)** A schematic representation of the positions of the detection channels (indicated by the green line) formed by the light sources and the detectors. **(C,D)** Schematic representations of the channel positions in the left **(C)** and right **(D)** hemispheres during fNIRS. Each gray dot represents the center of the detection channel.

fNIRS was performed using a multichannel continuous-wave near-infrared optical imaging system (Nirsmart, Huichuang, China) to record fNIRS data in continuous-wave mode, as described in previous studies (Bu et al., 2018; Li et al., 2020). fNIRS recordings were generated using NIRScan software (Version 2.3.5.1a, Huichuang, China). Based on the results reported by previous studies on vocal emotions in infants (Benavides-Varela et al., 2011; Minagawa-Kawai et al., 2011; Sato et al., 2012) and adults (Bruck et al., 2011; Fruhholz et al., 2016) with normal hearing, as well as the remodeling known to occur in the cortex after cochlear implantation, we placed the optodes over the temporal, frontal, and central regions of the brain, using an elastic cap with a 46–50 cm diameter (Huichuang, China), using the international 10–20 system (Okamoto et al., 2004; Figures 2A,B). We used 20 optical emitters (intensity greater than 50 mW/wavelength) and 16 two-wavelength (760 and 850 nm) detectors to form 52 available channels (26 per hemisphere), with the optical source and detector at a distance of approximately 2.50 cm (see Table 2 and Figures 2C,D).

**TABLE 2:** Channels and corresponding brain regions.

Channel	Brodmann area	*P*	Channel	Brodmann area	*P*
1	Middle temporal gyrus	0.825	27	Pre-motor and supplementary motor cortex	0.858
2	Inferior prefrontal gyrus	0.987	28	Dorsolateral prefrontal cortex	0.579
3	Middle temporal gyrus	0.791	29	Supramarginal gyrus part of Wernicke’s area	0.291
4	Inferior prefrontal gyrus	1	30	Supramarginal gyrus part of Wernicke’s area	1
5	Middle temporal gyrus	0.796	31	Dorsolateral prefrontal cortex	0.451
6	Pars triangularis Broca’s area	0.441	32	Dorsolateral prefrontal cortex	0.843
7	Middle temporal gyrus	0.822	33	Supramarginal gyrus part of Wernicke’s area	0.645
8	Pre-motor and supplementary motor cortex	0.473	34	Angular gyrus, part of Wernicke’s area	0.608
9	Fusiform gyrus	0.665	35	Pre-motor and supplementary motor cortex	0.315
10	Middle temporal gyrus	0.619	36	Pre-motor and supplementary motor cortex	0.739
11	Visual association cortex 3	0.487	37	Supramarginal gyrus part of Wernicke’s area	0.927
12	Supramarginal gyrus part of Wernicke’s area	0.399	38	Primary and auditory association cortex	0.616
13	Visual association cortex 3	0.579	39	Inferior prefrontal gyrus	0.886
14	Visual association cortex 3	0.732	40	Middle temporal gyrus	0.592
15	Dorsolateral prefrontal cortex	0.946	41	Inferior prefrontal gyrus	1
16	Dorsolateral prefrontal cortex	0.824	42	Middle temporal gyrus	0.806
17	Primary and auditory association cortex	0.396	43	Pars opercularis, part of Broca’s area	0.505
18	Pre-motor and supplementary motor cortex	0.706	44	Inferior prefrontal gyrus	0.8
19	Supramarginal gyrus part of Wernicke’s area	0.951	45	Middle temporal gyrus	1
20	Primary somatosensory cortex	0.318	46	Middle temporal gyrus	0.822
21	Pre-motor and supplementary motor cortex	0.656	47	Superior temporal Gyrus	0.816
22	Pre-motor and supplementary motor cortex	0.713	48	Middle temporal gyrus	0.876
23	Angular gyrus, part of Wernicke’s area	0.718	49	Fusiform gyrus	0.611
24	Angular gyrus, part of Wernicke’s area	0.508	50	Fusiform gyrus	0.526
25	Supramarginal gyrus part of Wernicke’s area	1	51	Visual association cortex 3	0.746
26	Primary somatosensory cortex	0.303	52	Fusiform gyrus	0.814

### fNIRS Data Preprocessing

Data preprocessing uses the Nirspark software package (version 6.12, Huichuang, China), run in MATLAB (R2017b, The MathWorks, United States). The following steps were used to preprocess the signal. (1) The task-unrelated time intervals were removed. (2) The task-unrelated artifacts were removed. (3) The light intensity was converted into optical density. (4) The data were band-pass filtered between 0.01 and 0.2 Hz to remove the task-unrelated effects of low-frequency drift and high-frequency neurophysiological noise. (5) Based on the Beer–Lambert law, the optical density was converted into oxyhemoglobin and deoxyhemoglobin concentrations. The hemodynamic response function initial time was set to -2 s, and the end time was set to 20 s (with “-2–0 s” as the reserved baseline state and “0–20 s” as the time for a single block paradigm). (6) With the “fear, angry, happy, neutral” duration set to 15 s, the oxyhemoglobin concentrations for each block paradigm were superimposed and averaged to generate a block average result. Because oxyhemoglobin is more sensitive to changes between conditions than deoxyhemoglobin and is typically associated with a better signal-to-noise ratio (Strangman et al., 2002), the subsequent statistical analysis only used oxyhemoglobin.

### Statistical Analyses

The preprocessed fNIRS data and behavioral data were counted using SPSS software (version 21.0, SPSS company, United States). We conducted vocal emotional stimulation (fearful, angry, happy, and neutral) by time (preoperative and postoperative) repeated-measures analysis of variance for each channel. To investigate the specific period during which the vocal emotion recognition ability was generated, a one-way analysis of variance was performed on those channels with significant main effects. Levene’s test was performed to detect the homogeneity of variance. When *p* > 0.05, variance was considered homogeneous. Follow-up analyses involved pairwise comparisons between the four emotional conditions, and the Bonferroni corrected method was used to correct for multiple comparisons between pairs. Pearson’s correlational analysis was used to measure the correlation between cortical responses to vocal emotional stimuli and behavioral results. For all analyses, *p* < 0.05 was considered significant.

## Results

### Behavioral Outcomes

**FIGURE 3:**
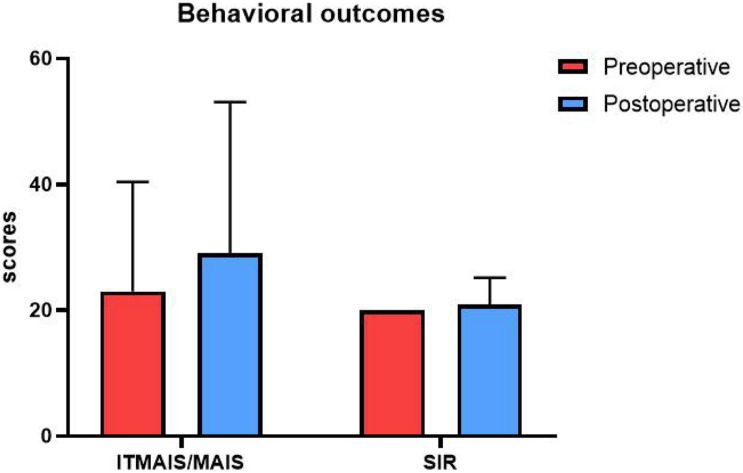
Behavioral outcomes. The vertical axis is the score, and the horizontal axis is the test type. Red represents the preoperative test score, and blue represents the postoperative test score. The scores of the two tests did not differ significantly between the two periods.

We performed a one-way analysis of variance on the two behavioral outcomes, the IT-MAIS/MAIS and SIR tests, comparing the preoperative and postoperative performance. In the preoperative test, the participants’ average score for the IT-MAIS/MAIS was 23.00, with a standard deviation of 17.41, and the average score for the SIR was 20, with a standard deviation of 0. In the postoperative test, the average score for the IT-MAIS/MAIS was 29.09, with a standard deviation of 24.00, and the average score for the SIR was 20.01, with a standard deviation of 4.26. No significant difference was identified between the behavioral results for either test (*p* > 0.05; Figure 3).

### fNIRS

**FIGURE 4:**
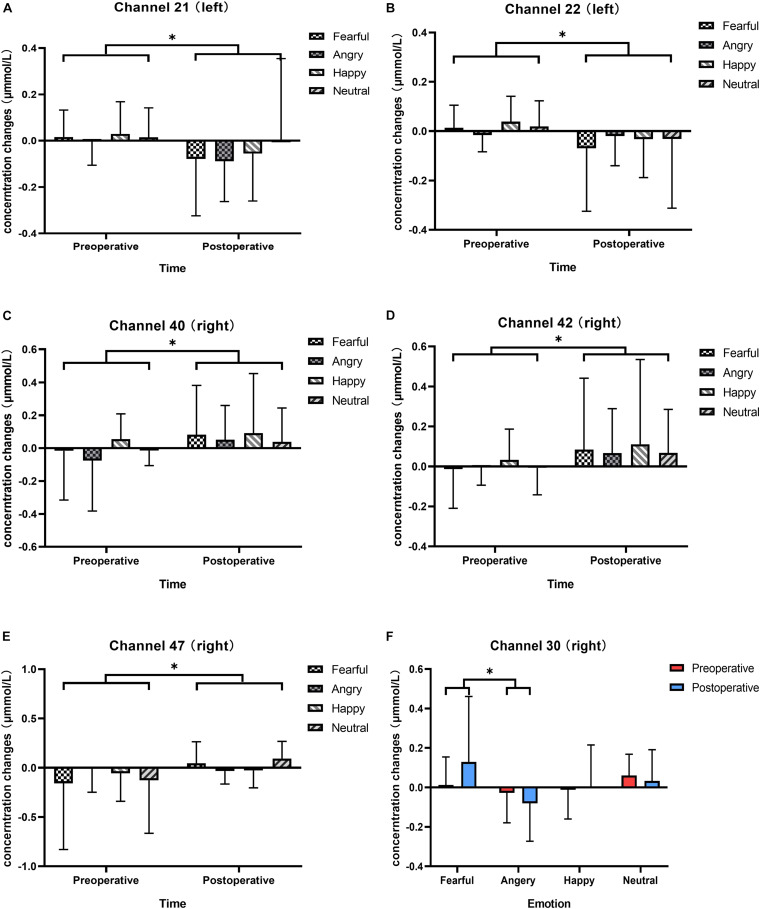
Changes in the responses of cortical oxyhemoglobin to four vocal emotional stimuli (the white grid pattern represents fearful emotion, the gray grid pattern represents angry emotion, the white diagonal pattern represents happy emotion, and the gray diagonal pattern represents neutral emotion) in the two-period test (red represents the preoperative test, blue represents the postoperative test). The six subplots show channels 21, 22 (located in the left pre-motor and supplementary motor cortex), 30 (located in the right supramarginal gyrus of Wernicke’s area), 40, 42 (located in the middle temporal gyrus), and 47 (located in the superior temporal gyrus). **(A,B)** In channels 21 and 22 (located in the left pre-motor and supplementary motor cortex), there is a significant difference in cortical activation elicited by vocal emotional stimulation in the preoperative and postoperative tests. **(C,D)** In channels 40 and 42 (located in the right middle temporal gyrus), there is a significant difference in cortical activation elicited by vocal emotional stimulation in the preoperative and postoperative tests. **(E)** In channel 47 (located in the right superior temporal gyrus), there is a significant difference in cortical activation elicited by vocal emotional stimulation in the preoperative and postoperative tests. **(F)** In channel 30 (located in the right supramarginal gyrus of Wernicke’ s area), cortical activation elicited by fear is significantly different than that elicited by anger. ^∗^*p* < 0.05.

We analyzed changes in the oxyhemoglobin response for each channel in response to four types of vocal emotional stimuli during two periods using a repeated-measures analysis of variance. The results showed that the main effect of the period factor (preoperative and postoperative) was significant for channels 21 (*F* = 5.970, *p* = 0.017, η^2^ = 0.066), 22 (*F* = 5.217, *p* = 0.025, η^2^ = 0.058), 40 (*F* = 4.829, *p* = 0.031, η^2^ = 0.054), 42 (*F* = 4.885, *p* = 0.030, η^2^ = 0.055), and 47 (*F* = 4.361, *p* = 0.040, η^2^ = 0.049; Figures 4A–E), whereas the main effect of vocal emotional stimulation (fearful, angry, happy, and neutral) was not significant (*F* < 0.792, *p* > 0.502, η^2^ < 0.028), and the interaction between vocal emotional stimulation and period was not significant (*F* < 1.418, *p* > 0.243, η^2^ < 0.048). However, in channel 30, the main effect of vocal emotional stimulation was significant (*F* = 3.122, *p* = 0.030, η^2^ = 0.100), whereas the main effect of the period was not significant (*F* = 0.220, *p* = 0.640, η^2^ = 0.003), and the interaction between vocal emotional stimulation and period was not significant (*F* = 2.072, *p* = 0.110, η^2^ = 0.069). Further pairwise comparison results showed that a significant difference between fear and anger (*p* = 0.038, Bonferroni corrected), and the cortical response when hearing a fear stimulus was greater than that hearing and anger stimulus (Figure 4F). The results of the one-way analysis of variance showed no significant difference in the response of channel 30 to the four vocal emotional stimuli in the preoperative test (*F* = 1.689, *p* = 0.176, η^2^ = 0.057), whereas a significant difference was identified in the postoperative test (*F* = 3.051, *p* = 0.033, η^2^ = 0.098). The cortical response to a fear stimulus was greater than that in response to an anger stimulus (*p* = 0.022, Bonferroni corrected). All statistical results were reported in Table 3.

**TABLE 3:** Statistical results of fNIRS test.

Channel	Emotion			Pre- and post-operation			Interaction		
	*F*	*p*	η^2^	*F*	*p*	η^2^	*F*	*p*	η^2^
1	0.096	0.962	0.003	3.461	0.066	0.040	0.680	0.567	0.024
2	0.814	0.490	0.028	0.287	0.594	0.003	0.378	0.769	0.013
3	1.016	0.390	0.035	0.003	0.955	< 0.001	0.526	0.666	0.018
4	0.648	0.586	0.023	1.232	0.270	0.014	0.868	0.461	0.030
5	0.066	0.978	0.002	1.251	0.267	0.015	0.268	0.848	0.009
6	1.142	0.337	0.039	1.600	0.209	0.019	0.483	0.695	0.017
7	1.407	0.246	0.048	0.685	0.410	0.008	1.047	0.376	0.036
8	1.536	0.211	0.052	1.008	0.318	0.012	1.875	0.140	0.063
9	0.599	0.617	0.021	0.203	0.653	0.002	0.353	0.787	0.012
10	0.986	0.404	0.034	0.265	0.608	0.003	0.936	0.427	0.032
11	0.559	0.644	0.020	0.422	0.518	0.005	0.316	0.814	0.011
12	2.278	0.085	0.075	1.282	0.261	0.015	0.912	0.439	0.032
13	2.149	0.100	0.071	0.087	0.769	0.001	0.231	0.874	0.008
14	0.613	0.608	0.021	0.033	0.857	< 0.001	0.516	0.673	0.018
15	1.979	0.123	0.066	0.267	0.607	0.003	0.566	0.639	0.020
16	1.593	0.197	0.054	2.023	0.159	0.024	1.301	0.280	0.044
17	1.137	0.339	0.039	0.144	0.705	0.002	0.815	0.489	0.028
18	0.545	0.653	0.019	0.093	0.762	0.001	0.542	0.655	0.019
19	0.686	0.563	0.024	1.199	0.277	0.014	2.236	0.090	0.074
20	0.653	0.583	0.023	0.262	0.610	0.003	0.476	0.700	0.017
21	0.545	0.653	0.019	5.970	0.017	0.066	0.454	0.715	0.016
22	0.264	0.851	0.009	5.217	0.025	0.058	0.575	0.633	0.020
23	0.218	0.884	0.008	0.471	0.494	0.006	0.375	0.772	0.013
24	1.862	0.142	0.062	0.071	0.791	0.001	0.343	0.795	0.012
25	0.810	0.492	0.028	0.082	0.775	0.001	0.167	0.918	0.006
26	0.398	0.755	0.014	2.188	0.143	0.025	0.593	0.621	0.021
27	0.863	0.464	0.030	0.651	0.422	0.008	0.516	0.673	0.018
28	0.340	0.796	0.012	0.031	0.862	< 0.001	0.584	0.627	0.020
29	1.176	0.324	0.040	0.154	0.696	0.002	0.839	0.476	0.029
30	3.122	0.030	0.100	0.220	0.640	0.003	2.072	0.110	0.069
31	1.454	0.233	0.049	2.582	0.112	0.030	1.197	0.316	0.041
32	0.033	0.992	0.001	2.580	0.112	0.030	0.571	0.636	0.020
33	2.083	0.109	0.069	< 0.001	0.988	< 0.001	1.495	0.222	0.051
34	2.528	0.063	0.083	0.012	0.915	< 0.001	0.615	0.607	0.021
35	2.175	0.097	0.072	1.220	0.273	0.014	0.643	0.589	0.022
36	1.954	0.127	0.065	0.383	0.538	0.005	0.465	0.708	0.016
37	2.351	0.078	0.077	0.319	0.574	0.004	0.291	0.832	0.010
38	0.140	0.936	0.005	0.326	0.569	0.004	0.195	0.899	0.007
39	1.319	0.274	0.045	0.753	0.388	0.009	0.526	0.666	0.018
40	0.792	0.502	0.028	4.829	0.031	0.054	0.338	0.798	0.012
41	0.690	0.561	0.024	0.032	0.858	< 0.001	0.477	0.699	0.017
42	0.270	0.847	0.010	4.885	0.030	0.055	0.028	0.994	0.001
43	0.130	0.942	0.005	0.385	0.537	0.005	0.685	0.564	0.024
44	1.431	0.240	0.049	1.013	0.317	0.012	0.906	0.442	0.031
45	1.109	0.350	0.038	0.330	0.567	0.004	1.048	0.376	0.036
46	1.343	0.266	0.046	1.950	0.166	0.023	0.498	0.685	0.017
47	0.107	0.956	0.004	4.361	0.040	0.049	1.418	0.243	0.048
48	0.258	0.856	0.009	0.153	0.697	0.002	0.878	0.456	0.030
49	1.859	0.143	0.062	0.066	0.797	0.001	2.405	0.073	0.079
50	1.898	0.136	0.063	0.062	0.804	0.001	2.020	0.117	0.067
51	0.120	0.948	0.004	2.624	0.109	0.030	0.624	0.601	0.022
52	0.436	0.728	0.015	0.031	0.860	< 0.001	0.965	0.413	0.033

### Neuro-Behavioral Correction

**FIGURE 5:**
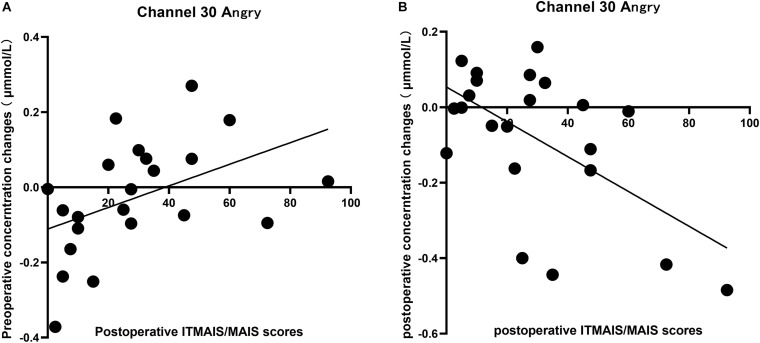
Correlation between the changes in the cortical oxyhemoglobin response to angry vocal emotions in channel 30 (located in the right supramarginal gyrus of Wernicke’s area) and the changes in the IT-MAIS/MAIS score. **(A)** A positive correlation was observed between preoperative neural responses to postoperative behavioral outcomes (Pearson’s correlation coefficient, *r* = 0.455, *p* = 0.033, *n* = 22). **(B)** A negative correlation was observed between the postoperative neural responses to postoperative behavioral outcomes (Pearson’s correlation coefficient, *r* = −0.576, *p* = 0.005, *n* = 22).

Significant differences in the cortical response to different vocal emotional stimuli were identified in channel 30; therefore, the cortical data from channel 30 in response to the four vocal emotional stimuli were used to perform correlation analysis with the behavioral outcomes. The results showed that changes in the neural response to anger observed in channel 30 were significantly correlated with the IT-MAIS/MAIS results. A positive correlation was identified between the preoperative cortical activation elicited by anger and the postoperative IT-MAIS/MAIS results (*r* = 0.455, *p* = 0.033, *n* = 22; Figure 5A). The results also showed a negative correlation between the postoperative cortical activation elicited by anger and the postoperative IT-MAIS/MAIS results (*r* = −0.576, *p* = 0.005, *n* = 22; Figure 5B). However, in response to other vocal emotional stimuli, no significant correlations with behavioral results were found (*p* > 0.05).

## Discussion

Although the perception and recognition of vocal emotions in neonates have been reported previously (Zhang et al., 2019), the present study is the first to focus on the perception and recognition of vocal emotions during the early period following CI operations in prelingual deaf infants and toddlers. We expected to describe the neural processing used in prelingual deaf infants and toddlers with vocal emotional stimuli and compared these before and early after surgery.

### Regional Differences in Cortical Activation Elicited by Vocal Emotions Before and After Hearing Reconstruction

The neural responses to vocal emotional stimuli in the preoperative and postoperative fNIRS tests were significantly different for the left channels 21 and 22 [located in (pre-)SMA] and the right channels 40, 42, and 47 (located in STG and MTG) in the present study (Figures 4A–E). The neural response of the left (pre-)SMA to vocal emotional stimulation changed from preoperative activation to postoperative inhibition. By contrast, the neural responses of the right MTG and STG to vocal emotional stimuli changed from preoperative inhibition to postoperative activation. To our best knowledge, this is the first observation to describe the neural processing of vocal emotional stimulation in prelingual deaf infants and toddlers, which revealed changes from the abnormal neural processing under hearing loss conditions to temporal auditory cortex processing after hearing reconstruction, which more closely resembled the normal processing locations. Previous studies have shown that the activity of the (pre-)SMA participates in emotional regulation initiated by the frontal lobe region and can affect the subcortical structures related to emotional regulation. The function of the (pre-)SMA is to reconceptualize and express information, which depends on language processing, memory tasks and embodied cognition (Kohn et al., 2014). The theory of embodied cognition and embodied emotion processing can help understand this result. The perceptual, motor, and emotional nervous systems are highly interconnected. The neuronal response of a system can reconnect neurons that were active during the initial experience through a cascade effect, reproducing the experience in multiple modes (Damasio, 1989; Wilson, 2002; Niedenthal, 2007). This theory suggests that the preoperative auditory emotional cognitive task processing is achieved by recruiting the SMA to reconceptualize the limited auditory perception information and activate the original neural state (obtained through a variety of channels, such as facial expressions, movements, and limited vocal emotional information) of the emotional information in memory. When postoperative hearing is reconstructed, the right MTG and STG will be recruited for the processing of vocal emotional cognitive tasks, which is in line with the lateralization theory of the brain, in which the right temporal area is the dominant area of emotion and prosody (Arimitsu et al., 2011; Güntürkün et al., 2020. Previous studies in people with normal hearing also observed a strong response for the right MTG and STG under vocal emotional stimulation (Zhang et al., 2017, 2019). These results show that the neural processing for vocal emotional stimulation in ITCIs gradually developed into a model similar to that observed in people with normal hearing. However, CI users have been described as having difficulty with the perception and recognition of vocal emotions compared with normal-hearing peers, which would impact the quality of life (Schorr et al., 2009).

### Differences in Vocal Emotion Recognition and Bias Before and After Hearing Reconstruction

In the preoperative fNIRS test, no significant difference in the neural response was identified for any area in response to the four vocal emotional stimuli. In the postoperative test, a significant difference was identified between the neural responses to fear and anger stimuli in channel 30 (located in the right SMG), and the response of fear was stronger than that of anger (Figure 4F). The activities of the SMG are related to the enhancement of the auditory perception of vocal emotion (Kochel et al., 2015). The results of previous studies on neonates with normal hearing also indicated that the right SMG responds strongly to fear (Zhang et al., 2017, 2019). Our results are not completely consistent with previous studies on neonates with normal hearing. Specifically, in our study, prelingual deaf ITCIs recognized fewer types of emotions than normal-hearing neonates, and differences in emotional neural responses only appeared in the right SMG, not in the right MTG or STG. Previous results have shown that at this very early stage, the participants are still in the process of remodeling from the abnormal neural processing to normal neural processing, similar to that observed in people with normal hearing. This also may be due to the limitations of CI itself, or the difference in vocal cues of anger and fear (Jiam et al., 2017). Under these conditions, ITCI’s still developing auditory system and brain may encounter greater difficulties in completing vocal emotional neural processing. Interestingly, although prelingual deaf ITCIs appear to be less well-developed than normal-hearing neonates in terms of vocal emotion recognition, they showed a negative bias toward vocal emotional stimuli. Previous studies performed in people with normal hearing suggested that the development of negative bias occurred at 6 months after birth (Farroni et al., 2007; Vaish et al., 2008; Peltola et al., 2009; Grossmann et al., 2010; Hoehl and Striano, 2010; Rigato et al., 2010). The result of this study may indicate that the development of emotional bias occurs independent of any specific sensory systems. The development of emotional bias in this study may benefit from facial or physical emotional stimulation, and multiple perception modalities might promote the development of emotional bias. This result supports the view of human infants’ differential treatment of environmental threats, which is considered to be a mechanism conducive to evolution (Vaish et al., 2008).

### Differences in Correlation Between Cortical Activation Elicited by Vocal Emotions Before and After Hearing Reconstruction and Behavioral Outcomes

Finally, we tested the correlation between the preoperative and postoperative neural response levels to the four emotions in channel 30 (located in the right SMG) and the preoperative and postoperative IT-MAIS/MAIS and SIR scores (Figure 5). We found that among the four emotions, only the neural response elicited by anger was significantly correlated with hearing ability (measured by IT-MAIS/MAIS which is reported by caregiver). The preoperative neural response was positively correlated with the postoperative hearing ability, whereas the postoperative neural response was negatively correlated with the postoperative hearing ability. We found that postoperative emotion recognition was elicited by an increased neural response to fear emotions and the inhibition of the neural response to anger emotions. This finding indicates that in the preoperative abnormal vocal emotional neural processing when the cortex is less strongly inhibited by anger stimuli, the abnormal processing is less well-developed, which improves the postoperative auditory ability. In the relatively normal vocal emotion neural processing after surgery, the enhanced inhibition of cortical response elicited by anger correlated with the better development of vocal emotion recognition abilities and better auditory function.

In this study, fNIRS was used to compare the differences between preoperative and postoperative vocal emotion discrimination ability and related neural mechanisms in prelingual deaf infants and toddlers. In addition, huge individual differences in the effects of CI were observed. Although the current preoperative examination can provide a basis for identifying CI candidates, no objective examination method exists to provide a reliable basis for how much these candidates can benefit from CI (Zhao et al., 2020). We also explored the possibility of using fNIRS as an objective, preoperative neuroimaging tool to monitor the early postoperative effects of CI prelingual deaf infants and toddlers.

## Limitations

This study has some limitations. First, the sample size included in this study was small, which makes studying the influences of differences in patient fixed factors that may exist within the group difficult to perform. In future studies, larger sample sizes are necessary to confirm our conclusions. Second, the detection range of fNIRS only includes the cortical areas and cannot show changes in activity among deeper structures. In future research, combinations of other neuroimaging methods that are suitable for repeated measurement will be necessary to study the origins of changes throughout the entire vocal emotional cognitive network both before and after CI implantation in prelingual deaf ITCIs. To observe the initial changes after cochlear implantation, this study selected vocal emotional stimulation, which requires less auditory experience and develops earlier than other auditory processing systems. Although studies have confirmed that the cognitive abilities of vocal emotional stimulation are related to future speech abilities, whether this correlation is stable remains unclear, and more long-term follow-up studies remain necessary to confirm this.

## Conclusion

In this study, we used fNIRS to monitor changes in neural processing for vocal emotional stimulation in prelingual deaf ITCIs before and after surgery. In addition, we explored the correlation between neural responses to vocal emotional stimulation and postoperative effects. The behavioral assessment showed no difference between the postoperative scores and the preoperative scores. However, significant differences between the preoperative and postoperative neural processing of the vocal emotional cognitive task were found during the neural assessment. A changing trend from the recruitment of the left (pre-)SMA during preoperative hearing loss to the recruitment of the right MTG and STG after hearing reconstruction was observed. We also found no significant difference in the response of each area to each emotional stimulus before the operation, whereas a negative bias was found in the right SMG after the operation. And there is a significantly stronger cortical response elicited by fear relative to anger that was identified after the operation. Finally, we found that the cortical response to anger in the right SMG was significantly correlated with the early CI behavioral results. Our research supplements the early stage data regarding the vocal emotion perception and recognition differences in CI users and broadens our horizons for future research in this field. This study provides more meaningful consultation suggestions for CI candidates and provides a basis for early and personalized rehabilitation strategies for CI users.

## Data Availability Statement

The raw data supporting the conclusions of this article will be made available by the authors, without undue reservation.

## Ethics Statement

The studies involving human participants were reviewed and approved by Ethical Committee of Peking University and the Peking University First Hospital. Written informed consent to participate in this study was provided by the participants’ legal guardian/next of kin. Written informed consent was obtained from the minor(s)’ legal guardian/next of kin for the publication of any potentially identifiable images or data included in this article.

## Author Contributions

YW: experimental design, data collection, data processing, and manuscript writing. LL: data processing. YZ, CW, TX, and QH: data collection. XH and YL: experimental design, test task writing, and project implementation management. All authors contributed to the article and approved the submitted version.

## Conflict of Interest

The authors declare that the research was conducted in the absence of any commercial or financial relationships that could be construed as a potential conflict of interest.

## Publisher’s Note

All claims expressed in this article are solely those of the authors and do not necessarily represent those of their affiliated organizations, or those of the publisher, the editors and the reviewers. Any product that may be evaluated in this article, or claim that may be made by its manufacturer, is not guaranteed or endorsed by the publisher.

## References

[B1] Ainsworth M. D. Bell S. M. (1970). Attachment, exploration, and separation: illustrated by the behavior of one-year-olds in a strange situation. *Child Dev.* 41 49–67. 5490680

[B2] Allen M. C. Nikolopoulos T. P. O’Donoghue G. M. (1998). Speech intelligibility in children after cochlear implanation. *Otol. Neurotol.* 19 742–746. 9831147

[B3] Anderson C. A. Wiggins I. M. Kitterick P. T. Hartley D. E. (2019). Pre-operative brain imaging using functional near-infrared spectroscopy helps predict cochlear implant outcome in deaf adults. *J. Assoc. Res. Otolaryngol.* 20 511–528. 31286300 10.1007/s10162-019-00729-z PMC6797684

[B4] Arimitsu T. Uchida-Ota M. Yagihashi T. Kojima S. Watanabe S. Hokuto I. (2011). Functional hemispheric specialization in processing phonemic and prosodic auditory changes in neonates. *Front. Psychol.* 2:202. 10.3389/fpsyg.2011.00202 21954386 PMC3173826

[B5] Benavides-Varela S. Gomez D. M. Mehler J. (2011). Studying neonates’ language and memory capacities with functional near-infrared spectroscopy. *Front. Psychol.* 2:64. 10.3389/fpsyg.2011.00064 21687439 PMC3110522

[B6] Bruck C. Kreifelts B. Wildgruber D. (2011). Emotional voices in context: a neurobiological model of multimodal affective information processing. *Phys. Life Rev.* 8 383–403. 10.1016/j.plrev.2011.10.002 22035772

[B7] Bryant G. Barrett H. C. (2008). Vocal emotion recognition across disparate cultures. *J. Cogn. Cult.* 8 135–148. 10.1163/156770908x289242

[B8] Bu L. Wang D. Huo C. Xu G. Li Z. Li J. (2018). Effects of poor sleep quality on brain functional connectivity revealed by wavelet-based coherence analysis using NIRS methods in elderly subjects. *Neurosci. Lett.* 668 108–114. 10.1016/j.neulet.2018.01.026 29353214

[B9] Cannon S. A. Chatterjee M. (2019). Voice emotion recognition by children with mild-to-moderate hearing loss. *Ear Hear.* 40 477–492. 10.1097/AUD.0000000000000637 30074504 PMC6373174

[B10] Cavicchiolo S. Mozzanica F. Guerzoni L. Murri A. Dall’Ora I. Ambrogi F. (2018). Early prelingual auditory development in Italian infants and toddlers analysed through the Italian version of the infant-toddler meaningful auditory integration scale (IT-MAIS). *Eur. Arch. Otorhinolaryngol.* 275 615–622. 10.1007/s00405-017-4847-6 29248951

[B11] Chatterjee M. Zion D. J. Deroche M. L. Burianek B. A. Limb C. J. Goren A. P. (2015). Voice emotion recognition by cochlear-implanted children and their normally-hearing peers. *Hear. Res.* 322 151–162. 10.1016/j.heares.2014.10.003 25448167 PMC4615700

[B12] Cheng Y. Lee S. Y. Chen H. Y. Wang P. Y. Decety J. (2012). Voice and emotion processing in the human neonatal brain. *J. Cogn. Neurosci.* 24 1411–1419. 10.1162/jocn_a_00214 22360593

[B13] Cooper R. P. Aslin R. N. (1989). The language environment of the young infant: implications for early perceptual development. *Can. J. Psychol.* 43 247–265. 10.1037/h0084216 2486498

[B14] Damasio A. R. (1989). Time-locked multiregional retroactivation: a systems-level proposal for the neural substrates of recall and recognition. *Cognition* 33 25–62. 10.1016/0010-0277(89)90005-x 2691184

[B15] Denham S. A. McKinley M. Couchoud E. A. Holt R. (1990). Emotional and behavioral predictors of preschool peer ratings. *Child Dev.* 61 1145–1152. 2209184

[B16] Drotar D. Sturm L. (1988). Prediction of intellectual development in young children with early histories of nonorganic failure-to-thrive. *J. Pediatr. Psychol.* 13 281–296. 10.1093/jpepsy/13.2.281 3171820

[B17] Editorial Board of Chinese Journal of Otorhinolaryngology Head and Neck Surgery, Chinese Medical Association Otorhinolaryngology Head and Neck Surgery Branch, and Professional Committee of Hearing and Speech Rehabilitation of China Disabled Rehabilitation Association (2014). Guidelines for cochlear implant work (2013). *Chin. J. Otorhinolaryngol. Head Neck Surg.* 49 89–95. 10.3760/cma.j.issn.1673-0860.2014.02.001 24742505

[B18] Eisenberg N. Spinrad T. L. Eggum N. D. (2010). Emotion-related self-regulation and its relation to children’s maladjustment. *Annu. Rev. Clin. Psychol.* 6 495–525. 10.1146/annurev.clinpsy.121208.131208 20192797 PMC3018741

[B19] Farroni T. Menon E. Rigato S. Johnson M. H. (2007). The perception of facial expressions in newborns. *Eur. J. Dev. Psychol.* 4 2–13. 10.1080/17405620601046832 20228970 PMC2836746

[B20] Fruhholz S. Grandjean D. (2013). Multiple subregions in superior temporal cortex are differentially sensitive to vocal expressions: a quantitative meta-analysis. *Neurosci. Biobehav. Rev.* 37 24–35. 10.1016/j.neubiorev.2012.11.002 23153796

[B21] Fruhholz S. Trost W. Kotz S. A. (2016). The sound of emotions-towards a unifying neural network perspective of affective sound processing. *Neurosci. Biobehav. Rev.* 68 96–110. 10.1016/j.neubiorev.2016.05.002 27189782

[B22] Geers A. E. Davidson L. S. Uchanski R. M. Nicholas J. G. (2013). Interdependence of linguistic and indexical speech perception skills in school-age children with early cochlear implantation. *Ear Hear.* 34 562–574. 10.1097/AUD.0b013e31828d2bd6 23652814 PMC3740036

[B23] Glenn S. M. Cunningham C. C. (1983). What do babies listen to most? A developmental study of auditory preferences in nonhandicapped infants and infants with Down’s syndrome. *Dev. Psychol.* 19 332–337. 10.1037/0012-1649.19.3.332 6459215

[B24] Gomez D. M. Berent I. Benavides-Varela S. Bion R. A. Cattarossi L. Nespor M. (2014). Language universals at birth. *Proc. Natl. Acad. Sci. U.S.A.* 111 5837–5841. 10.1073/pnas.1318261111 24706790 PMC4000834

[B25] Gottlieb G. (1971). *Ontogenesis of Sensory Function in Birds and Mammals.* New York, NY: Academic Press.

[B26] Grossmann T. Oberecker R. Koch S. P. Friederici A. D. (2010). The developmental origins of voice processing in the human brain. *Neuron* 65 852–858. 10.1016/j.neuron.2010.03.001 20346760 PMC2852650

[B27] Güntürkün O. Ströckens F. Ocklenburg S. (2020). Brain lateralization: a comparative perspective. *Physiol. Rev.* 100 1019–1063. 10.1152/physrev.00006.2019 32233912

[B28] Hoehl S. Striano T. (2010). The development of emotional face and eye gaze processing. *Dev. Sci.* 13 813–825. 10.1111/j.1467-7687.2009.00944.x 20977553

[B29] Jiam N. T. Caldwell M. Deroche M. L. Chatterjee M. Limb C. J. (2017). Voice emotion perception and production in cochlear implant users. *Hear. Res.* 352 30–39. 10.1016/j.heares.2017.01.006 28088500 PMC5937709

[B30] Jiam N. T. Limb C. (2020). Music perception and training for pediatric cochlear implant users. *Expert Rev. Med. Devices* 17 1193–1206. 10.1080/17434440.2020.1841628 33090055

[B31] Kang S. Y. Colesa D. J. Swiderski D. L. Su G. L. Raphael Y. Pfingst B. E. (2010). Effects of hearing preservation on psychophysical responses to cochlear implant stimulation. *J. Assoc. Res. Otolaryngol.* 11 245–265. 19902297 10.1007/s10162-009-0194-7 PMC2862914

[B32] Kasatkin N. I. (1972). First conditioned reflexes and the beginning of the learning process in the human infant. *Adv. Psychobiol.* 1 213–257. 4666669

[B33] Kochel A. Schongassner F. Feierl-Gsodam S. Schienle A. (2015). Processing of affective prosody in boys suffering from attention deficit hyperactivity disorder: a near-infrared spectroscopy study. *Soc. Neurosci.* 10 583–591. 10.1080/17470919.2015.1017111 25721229

[B34] Kohn N. Eickhoff S. B. Scheller M. Laird A. R. Fox P. T. Habel U. (2014). Neural network of cognitive emotion regulation–an ALE meta-analysis and MACM analysis. *Neuroimage* 87 345–355. 10.1016/j.neuroimage.2013.11.001 24220041 PMC4801480

[B35] Kong Y.-Y. Mullangi A. Marozeau J. Epstein M. (2011). Temporal and spectral cues for musical timbre perception in electric hearing. *J. Speech Lang. Hear. Res.* 54 981–994. 21060140 10.1044/1092-4388(2010/10-0196) PMC3107380

[B36] Li Q. Feng J. Guo J. Wang Z. Li P. Liu H. (2020). Effects of the multisensory rehabilitation product for home-based hand training after stroke on cortical activation by using NIRS methods. *Neurosci. Lett.* 717:134682. 10.1016/j.neulet.2019.134682 31837442

[B37] Linnankoski I. Leinonen L. Vihla M. Laakso M.-L. Carlson S. (2005). Conveyance of emotional connotations by a single word in English. *Speech Commun.* 45 27–39. 10.1016/j.specom.2004.09.007

[B38] Liu P. Pell M. D. (2012). Recognizing vocal emotions in Mandarin Chinese: a validated database of Chinese vocal emotional stimuli. *Behav. Res. Methods* 44 1042–1051. 10.3758/s13428-012-0203-3 22539230

[B39] Lu X. Qin Z. (2018). Auditory and language development in Mandarin-speaking children after cochlear implantation. *Int. J. Pediatr. Otorhinolaryngol.* 107 183–189. 10.1016/j.ijporl.2018.02.006 29501303

[B40] Ludlow A. Heaton P. Rosset D. Hills P. Deruelle C. (2010). Emotion recognition in children with profound and severe deafness: do they have a deficit in perceptual processing? *J. Clin. Exp. Neuropsychol.* 32 923–928. 10.1080/13803391003596447 20349386

[B41] Minagawa-Kawai Y. van der Lely H. Ramus F. Sato Y. Mazuka R. Dupoux E. (2011). Optical brain imaging reveals general auditory and language-specific processing in early infant development. *Cereb. Cortex* 21 254–261. 10.1093/cercor/bhq082 20497946 PMC3020578

[B42] Most T. Michaelis H. (2012). Auditory, visual, and auditory–visual perceptions of emotions by young children with hearing loss versus children with normal hearing. *J. Speech Lang. Hear. Res.* 55 1148–1162. 10.1044/1092-4388(2011/11-0060) 22271872

[B43] Most T. Weisel A. Zaychik A. (1993). Auditory, visual and auditory-visual identification of emotions by hearing and hearing-impaired adolescents. *Br. J. Audiol.* 27 247–253. 10.3109/03005369309076701 8312847

[B44] Niedenthal P. M. (2007). Embodying emotion. *Science* 316 1002–1005. 10.1126/science.1136930 17510358

[B45] Okamoto M. Dan H. Sakamoto K. Takeo K. Shimizu K. Kohno S. (2004). Three-dimensional probabilistic anatomical cranio-cerebral correlation via the international 10-20 system oriented for transcranial functional brain mapping. *Neuroimage* 21 99–111. 10.1016/j.neuroimage.2003.08.026 14741647

[B46] Pegg J. E. Werker J. F. McLeod P. J. (1992). Preference for infant-directed over adult-directed speech: evidence from 7-week-old infants. *Infant Behav. Dev.* 15 325–345. 10.1016/0163-6383(92)80003-d

[B47] Pell M. D. Monetta L. Paulmann S. Kotz S. A. (2009). Recognizing emotions in a foreign language. *J. Nonverbal Behav.* 33 107–120.

[B48] Peltola M. J. Leppanen J. M. Maki S. Hietanen J. K. (2009). Emergence of enhanced attention to fearful faces between 5 and 7 months of age. *Soc. Cogn. Affect. Neurosci.* 4 134–142. 10.1093/scan/nsn046 19174536 PMC2686224

[B49] Piper S. K. Krueger A. Koch S. P. Mehnert J. Habermehl C. Steinbrink J. (2014). A wearable multi-channel fNIRS system for brain imaging in freely moving subjects. *Neuroimage* 85(Pt 1) 64–71. 10.1016/j.neuroimage.2013.06.062 23810973 PMC3859838

[B50] Planalp S. (1996). Varieties of cues to emotion in naturally occurring situations. *Cogn. Emot.* 10 137–154.

[B51] Rigato S. Farroni T. Johnson M. H. (2010). The shared signal hypothesis and neural responses to expressions and gaze in infants and adults. *Soc. Cogn. Affect. Neurosci.* 5 88–97. 10.1093/scan/nsp037 19858107 PMC2840839

[B52] Saliba J. Bortfeld H. Levitin D. J. Oghalai J. S. (2016). Functional near-infrared spectroscopy for neuroimaging in cochlear implant recipients. *Hear. Res.* 338 64–75. 10.1016/j.heares.2016.02.005 26883143 PMC4967399

[B53] Sato H. Hirabayashi Y. Tsubokura H. Kanai M. Ashida T. Konishi I. (2012). Cerebral hemodynamics in newborn infants exposed to speech sounds: a whole-head optical topography study. *Hum. Brain Mapp.* 33 2092–2103. 10.1002/hbm.21350 21714036 PMC6870359

[B54] Sauter D. A. Eisner F. Calder A. J. Scott S. K. (2010). Perceptual cues in nonverbal vocal expressions of emotion. *Q. J. Exp. Psychol. (Hove)* 63 2251–2272. 10.1080/17470211003721642 20437296 PMC4178283

[B55] Schorr E. A. Roth F. P. Fox N. A. (2009). Quality of life for children with cochlear implants: perceived benefits and problems and the perception of single words and emotional sounds. *J. Speech Lang. Hear. Res.* 52 141–152. 10.1044/1092-4388(2008/07-0213) 18664684

[B56] Strangman G. Culver J. P. Thompson J. H. Boas D. A. (2002). A quantitative comparison of simultaneous BOLD fMRI and NIRS recordings during functional brain activation. *Neuroimage* 17 719–731. 10.1006/nimg.2002.1227 12377147

[B57] Thompson W. F. Balkwill L.-L. (2006). Decoding speech prosody in five languages. *Semiotica* 2006 407–424.

[B58] Trainor L. J. Austin C. M. Desjardins R. N. (2000). Is infant-directed speech prosody a result of the vocal expression of emotion? *Psychol. Sci.* 11 188–195. 10.1111/1467-9280.00240 11273402

[B59] Vaish A. Grossmann T. Woodward A. (2008). Not all emotions are created equal: the negativity bias in social-emotional development. *Psychol. Bull.* 134 383–403. 10.1037/0033-2909.134.3.383 18444702 PMC3652533

[B60] Wauters L. N. Knoors H. (2008). Social integration of deaf children in inclusive settings. *J. Deaf Stud. Deaf Educ.* 13 21–36. 10.1093/deafed/enm028 17573356

[B61] Wiefferink C. H. Rieffe C. Ketelaar L. De Raeve L. Frijns J. H. (2013). Emotion understanding in deaf children with a cochlear implant. *J. Deaf Stud. Deaf Educ.* 18 175–186. 10.1093/deafed/ens042 23232770

[B62] Wilson M. (2002). Six views of embodied cognition. *Psychon. Bull. Rev.* 9 625–636. 10.3758/bf03196322 12613670

[B63] Xu L. Zhou N. Chen X. Li Y. Schultz H. M. Zhao X. (2009). Vocal singing by prelingually-deafened children with cochlear implants. *Hear. Res.* 255 129–134. 19560528 10.1016/j.heares.2009.06.011 PMC2744154

[B64] Yang F. Zhao F. Zheng Y. Li G. (2020). Modification and verification of the infant-toddler meaningful auditory integration scale: a psychometric analysis combining item response theory with classical test theory. *Health Qual. Life Outcomes* 18:367. 10.1186/s12955-020-01620-9 33187553 PMC7663878

[B65] Zeng F. G. Rebscher S. Harrison W. Sun X. Feng H. (2008). Cochlear implants: system design, integration, and evaluation. *IEEE Rev. Biomed. Eng.* 1 115–142. 10.1109/RBME.2008.2008250 19946565 PMC2782849

[B66] Zhang D. Chen Y. Hou X. Wu Y. J. (2019). Near-infrared spectroscopy reveals neural perception of vocal emotions in human neonates. *Hum. Brain Mapp.* 40 2434–2448. 10.1002/hbm.24534 30697881 PMC6865553

[B67] Zhang D. Zhou Y. Hou X. Cui Y. Zhou C. (2017). Discrimination of emotional prosodies in human neonates: a pilot fNIRS study. *Neurosci. Lett.* 658 62–66. 10.1016/j.neulet.2017.08.047 28842278

[B68] Zhao C. Chronaki G. Schiessl I. Wan M. W. Abel K. M. (2019). Is infant neural sensitivity to vocal emotion associated with mother-infant relational experience? *PLoS One* 14:e0212205. 10.1371/journal.pone.0212205 30811431 PMC6392422

[B69] Zhao E. E. Dornhoffer J. R. Loftus C. Nguyen S. A. Meyer T. A. Dubno J. R. (2020). Association of patient-related factors with adult cochlear implant speech recognition outcomes: a meta-analysis. *JAMA Otolaryngol. Head Neck Surg.* 146 613–620. 32407461 10.1001/jamaoto.2020.0662 PMC7226297

[B70] Zimmerman-Phillips S. Osberger M. Robbins A. (1997). *Infant-Toddler: Meaningful Auditory Integration Scale (IT-MAIS).* Sylmar, LA: Advanced Bionics Corporation.

